# Transmission of genital human papillomavirus infection in couples: a population-based cohort study in rural China

**DOI:** 10.1038/srep10986

**Published:** 2015-07-23

**Authors:** Mengfei Liu, Zhonghu He, Chanyuan Zhang, Fangfang Liu, Ying Liu, Jingjing Li, Zhongyao Xu, Qiyan Wang, Dong Hang, Na Shen, Yaqi Pan, Chuanhai Guo, Hong Cai, Yang Ke

**Affiliations:** 1 Key laboratory of Carcinogenesis and Translational Research (Ministry of Education), Laboratory of Genetics, Peking University Cancer Hospital & Institute, No. 52 Fucheng Road, Haidian District, Beijing 100142, P.R. China

## Abstract

HPV transmission dynamics have rarely been studied in the general population, especially in China. We followed the genital HPV infection status of both partners in 874 couples aged 25-65 years from rural China for up to 7 bi-annual visits during 2009-2013. The positive HPV concordance and transmission rate for partners in a couple were evaluated and relevant risk factors were assessed. The concordance of any, oncogenic, and non-oncogenic HPV was 15.52%, 16.18% and 10.41%, respectively. Male-to-female transmission rate was 7.11, 12.13 and 4.77/1000 person months for any, oncogenic and non-oncogenic HPV respectively. The female-to-male transmission rate was 5.56, 2.37, and 17.01/1000 person months for any, oncogenic and non-oncogenic HPV respectively. The risk of male-to-female transmission was significantly higher than that of female-to-male transmission for oncogenic types. However, for non-oncogenic types, the risk of male-to-female transmission was significantly lower than that of female-to-male transmission. Younger couples, persistent infection with HPV, higher numbers of sexual partners and higher frequency of sexual intercourse were positively associated with HPV transmission in couples. Our results indicate that men in rural China play a more important role than men in western populations as a source of cervical oncogenic HPV infection in women.

HPV infection has been demonstrated to be the causative agent of cervical cancer in women, and it also plays an important role in anogenital and oropharyngeal cancers^1^. Direct genital mucosa contact during sexual intercourse is the principal route of HPV transmission^2^. Case-control studies have shown that HPV infections in men can increase the risk of cervical cancer in their partners^2^,^3^,^4^ as a reservoir of HPV^2^. Understanding the transmission dynamics of HPV infection in sexual partners is crucial for primary prevention of cervical cancer as well as other HPV related diseases in women and men, particularly as prophylactic vaccine will be introduced in mainland China^5^ and cost-effective strategies must be developed. Study of couples is an effective means for investigating how and to what extent men influence HPV infection in female sexual partners and vice versa. However, due to practical concerns, only 5 prospective studies concerning heterosexual transmission of HPV in couples have been carried out to date^6^,^7^,^8^,^9^,^10^, with sample sizes ranging from 25 to 486 couples. Three studies adopted 4-6 months follow-up intervals^6^,^7^,^8^, while two studies used much shorter intervals of 48 hours to 6 weeks^9^ and 2 months^10^. In all five studies, the rate of female-to-male transmission was higher than male-to-female transmission rate^6^,^7^,^8^,^9^,^10^. A decreasing trend in the point estimations of transmission rate associated with longer follow-up interval was observed across these studies. In some studies, defects in study design such as inadequate sample size, convenient sampling source, insufficient length of follow-up, and relatively narrow age range may have limited the reliability of conclusions and the comparability among studies^11^.A large population-based longitudinal study in healthy couples with a wide age range using rigorously standardized follow-up would better serve to improve our knowledge of HPV transmission dynamics in the general population.

In this study we followed genital HPV infection status in 874 couples aged 25 to 65 for up to 7 bi-annual visits from 2009 to 2013 by making use of an ongoing population-based cohort study in rural Anyang, China^12^. The aims of this study were to determine the type-specific HPV concordance and transmission rates in couples from the general population of rural China, and to evaluate potential risk factors for HPV transmission.

## Methods

### Study subjects

In 2007–2009 a population-based esophageal cancer cohort study (AECCS) was initiated in rural Anyang, China^12^. Briefly, 9 villages of rural Anyang were included as target villages based on the cluster sampling method and a relatively high response proportion (8638/10772 eligible subjects) was achieved in the baseline investigation. The current study utilizes a sub-cohort which includes 3 of 9 target villages, and the baseline investigation for this study was conducted concurrently with the 2^nd^ cross-section investigation of the AECCS in 2009-2010. The eligibility criteria for couples included in the current study were as follows: 1) being married and permanent resident in the target villages; 2) age 25-65 years; 3) female partner had an intact uterus and was not pregnant during the study period; 4) no history of cancer, cardiovascular disease or mental disorder; 5) no history of infection with HBV, HCV, or HIV; and 6) willingness to cooperate with the follow up and provide informed consent. All eligible couples were enrolled from 2009 to 2010 and were assessed bi-annually, for up to 7 visits for husbands and up to 6 visits for wives. Centralized examinations were conducted, and all couples were sampled within a period of two weeks at each follow-up visit. Study visits for male and female partners were conducted at the same visit.

### Specimen and data collection

For males, exfoliated cells from the penile shaft, glans penis (prepuce was not included), coronal sulcus, and scrotum were collected by an experienced doctor using saline-soaked swabs at each evaluation^13^. For females exfoliated cells from the cervical os and ectocervix were collected by a gynecologist using saline-soaked swabs. Each swab was placed in a vial containing 0.9% saline solution. All vials were centrifuged at 5000 rpm for 5 min and supernatants were discarded. Specimens were stored at −20 °C and subsequently at −70 °C pending DNA extraction and HPV detection. As described elsewhere^12^, a computer aided questionnaire interview was completed by all subjects during the baseline investigation to obtain the data on demographic characteristics and potential risk factors for HPV infection and transmission. To minimize the investigation bias for sensitive questions (e.g. sexual activity), we established a “standard protocol” for face-to-face interviews: 1) the interview was conducted one-on-one in a private room hosted by a well-trained interviewer with a medical background capable of speaking the local dialect, 2) before the interview, the interviewer introduced himself (herself) to the respondent first, and informed the respondent what information would be collected, and making it clear that the individual being interviewed may ask questions or elect not to answer given questions, 3) the interviewer read each question to the respondent and input his (her) answers into the survey system. Standardized explanations regarding the items in the questionnaire were provided if the respondent could not understand or respond correctly.

### Ethics statement

Research protocols and materials were approved by the Institutional Review Board of the Peking University School of Oncology, China. All methods were carried out according to the approved guidelines. All participants in this study provided informed written consent.

### Laboratory procedures

Specimen DNA was extracted using the Biomek 3000 workstation (Beckman Coulter, Brea, CA, USA). The β-globin gene was first evaluated by PCR, and only β-globin positive specimens were subjected to HPV evaluation using a pair of SPF1/GP6+ primers^14^. Sanger sequencing was used for genotyping to evaluate the HPV types, as described previously^13^,^15^,^16^. Samples with ambiguous HPV typing signals were subjected to further cloning and sequencing for multiple infections. The HPV types classified as oncogenic in this study were HPV-16, 18, 31, 33, 35, 39, 45, 51, 52, 56, 58, 59, 66 and 68^17^,^18^, and all other types including HPV-2, 3, 6, 7, 10, 11, 26, 27, 29, 30, 32, 40, 42, 43, 53, 54, 57, 62, 67, 69, 70, 74, 75, 81, 82, 83, 84, 87, 89, 90, 91, and 94 were classified as non-oncogenic types.

### Statistical analysis

For concordance analysis, only positive concordance of HPV on the basis of a type-specific principle was evaluated. Any couple meeting the conditions: a) both male and female provided ≥2 valid HPV results within the study period; b) either partner had at least 1 HPV positive visit, was included in the analysis. HPV concordance was defined as the proportion of co-positive couples for a specific HPV type out of all HPV exposed couples. For groups of genotypes (i.e. any HPV type, oncogenic and non-oncogenic HPV), concordance was defined as the proportion of couples co-positive for at least one of any type HPV, 14 oncogenic HPVs and 32 non-oncogenic HPVs. The Chi-square test was performed for specific HPV types to determine whether the observed concordant events significantly exceeded the expected value^19^. For groups of HPV, a Monte-Carlo simulation method was used for comparison of observed events and expected values^20^.

Analysis of male-to-female and female-to-male transmission was conducted separately under a type-specific principle. The infection was treated as the calculating unit and multiple infections in one participant were counted as separate events. In this study, we observed that the transmission event was far less likely to occur after the infection in the partner in whom HPV infection originated had been cleared (data not shown). In view of this fact, we employed an “HPV transmission window” to define the transmission event and calculate the person-time at risk of HPV transmission in couples. The “transmission window” opens at the time of the visit when one partner in a couple is found to be positive for a specific type of HPV for the first time, while his/her partner has been negative for the corresponding HPV type from enrollment to the time of that visit. The window closes at the earliest occurrence of any one of the following: a) the adjacent visit subsequent to the last positive visit of this infection in the HPV index partner, b) a type-specific transmission event occurs, c) the follow-up of the at-risk partner right censored. An HPV transmission event was defined as newly acquired type-specific infection in the at-risk partner within the transmission window. A couple was included in the transmission analysis if the at-risk partner had at least 2 consecutive visits within the “HPV transmission window”. Transmission rates were then calculated by dividing the number of transmission events by the total number of person months of transmission windows, and 95% CIs were estimated based on Poisson distribution^21^. Transmission rates of grouped HPV types were calculated by summing up the transmission infections of the relevant HPV types, with use of the jackknife method for adjustment of 95% CIs. For comparison of male-to-female versus female-to-male HPV transmission rates, a univariate Cox model including all exposed HPV infections with a sex indicator in the model, implemented with the Wei Lin Weissfeld (WLW) marginal model approach was used^22^.

Factors for HPV transmission evaluated in the current study included average age, average lifetime number of sexual partners and sexual frequency per week of study couples, as well as the level of education, type of job and presence of persistent HPV infection in a given couple. Persistent HPV infection was defined as ≥3 consecutive positive visits for the HPV-exposed partner in this study. Factors associated with HPV concordance were assessed using a logistic regression model, in which the Generalized Estimating Equation (GEE) was used to take into consideration of within subject correlation^23^. The WLW Cox model was used to assess these potential factors for HPV transmission.Single missing data points for HPV status were imputed using the “prior observation carried backward” approach as previously described^24^.

All statistical analyses were performed using STATA version 11.2 (STATA Corporation, College Station, TX, USA). Tests were two sided, and had a significance level of 0.05.

## Results

### Number of valid couples and follow up duration

In this study, 93.4% and 98.3% of genital exfoliated cell specimens from men and women were positive for the human beta-globin gene and were thus considered adequate for subsequent HPV testing. A total of 874 couples provided at least one valid HPV positive result for both partners, among whom HPV positivity was found in 749 of 4480 visits (16.7%) in men, and 345 of 4215 visits (8.2%) in women during the study period. The proportion of multiple infection was 8.5% in men (64/749) and 8.7% in women (30/345). Among these 874 couples, 406 (46.5%) HPV exposed couples provided at least 2 valid specimens from both partners and were included in the concordance evaluation. The median follow-up time was 33.6 months for males (IQR 18.8-38.0) and 30.4 months for females (IQR 23.8-31.9). For transmission analysis, 296 HPV exposed couples in whom HPV transmission had potential to occur within the defined transmission window were included with a median follow-up time of 35.7 months for males (IQR 19.4-38.3) and 30.5 months for females (IQR 25.1-32.2).

### Demographic and behavior characteristics in couples

The median age of both male and female subjects was 44 among these 874 couples as shown in Table 1. Most couples had an education level of junior middle school or below. Around two-thirds of the males worked in local and outside areas at the time of enrollment, while the majority of females (88.3%) were engaged in farming at home. Regarding behavioral factors, more males than females (11.1% vs. 0.8%, *p *< 0.001) reported having multiple sexual partner. Around half of these couples (50.5% for males and 60.3% for females) reported having sexual intercourse ≤1 each week. Persistent HPV infections were detected more frequently in males (15.8% vs. 4.7%, *p *< 0.001). No appreciable differences were observed in couples included in or not included in the concordance analysis and the transmission analysis, except that greater numbers of lifetime sexual partners were observed in couples included in the concordance analysis as compared with excluded couples (*p *= 0.013)(Table 1).

### HPV concordance within couples

The concordance of any HPV type, oncogenic and non-oncogenic HPV infection in couples was 15.52%, 16.18% and 10.41% respectively. The observed concordant events for most HPV types significantly exceeded the expected value. The concordance of oncogenic HPV was higher than non-oncogenic HPV, although the difference did not reach a significant level (*p *= 0.054, for oncogenic vs. non-oncogenic). Specifically, concordance for HPV-16, HPV-18, HPV-11 and HPV-6 was 12.17%, 5.45%, 22.22% and 42.86%, respectively (Table 2).

### HPV transmission rate within couples

Male-to-female transmission rates for any HPV type, oncogenic, non-oncogenic HPV and HPV-16/18/11/6 infection were 7.11, 12.13, 4.77 and 10.48 per 1000 PM respectively as shown in Table 3. The transmission rate of oncogenic types was significantly higher than non-oncogenic types (*p *= 0.011) in male-to-female transmission. The female-to-male transmission rates for any HPV type, oncogenic, non-oncogenic HPV and HPV-16/18/11/6 infection were 5.56, 2.37, 17.01 and 2.73 per 1000 PM, respectively. The transmission rate of oncogenic types was significantly lower than non-oncogenic types (*p* = 0.012) in female-to-male transmission.

For any type HPV, no significant difference was observed in the risk of male-to-female transmission versus female-to-male. However, the male-to-female transmission rate was significantly higher than the female-to-male transmission rate in oncogenic HPV types (Hazard ratio_M-to-F vs. F-to-M_ = 4.89, 95%CI: 1.36-17.55; *p *= 0.015), but the male-to-female transmission rate was significantly lower than the female-to-male transmission rate in non-oncogenic HPV types (Hazard ratio _M-to-F vs. F-to-M _= 0.27, 95%CI: 0.10-0.76; *p *= 0.013). For vaccination types, the risk of male-to-female transmission was higher than that of female-to-male transmission, but this difference did not reach a significant level (Hazard ratio _M-to-F vs. F-to-M_ = 3.69, 95%CI: 0.79-17.11; *p *= 0.096) (Table 3).

### Risk factors for HPV concordance and transmission within couples

For factor analyses, a significantly lower risk of concordant infection for both any and oncogenic HPV was observed in older couples aged 46-65 years, as compared with couples aged 25-45. Persistent HPV infection was consistently associated with an increased risk of both HPV concordance and transmission. Couples who reported having >1 lifetime sexual partner had a significantly elevated risk of HPV concordance in any and oncogenic HPV, and also had a significantly elevated risk of any type of HPV transmission. Risk of both HPV concordance and transmission in non-oncogenic types was significantly increased in couples who reported having 2 episodes of sexual intercourse per week as compared to couples who reported having sexual intercourse ≤1 per week (Table 4).

## Discussion

In this study, we investigated the positive concordance and transmission rate of genital HPV infection among 874 couples aged 25-65 from northern rural China with population-based sampling. We found that observed concordance in the majority of HPV types was significantly higher than expected. The risk of male-to-female transmission was around 4 times higher than that of female-to-male transmission for oncogenic HPV, and conversely, the male-to-female transmission rate of non-oncogenic HPV was 73% lower than that of female-to-male transmission.

Until now only 5 prospective studies have investigated HPV transmission in heterosexual couples. All these studies^6^,^7^,^8^,^9^,^10^ reported a significantly higher female-to-male transmission rate of any type of HPV than that of male-to-female transmission. Explanations for this finding included a) men acquire more transient infections^25^; b) HPV viral load is lower in men^19^,^26^,^27^; and c) men have a lower sero-conversion rate for HPV infection^28^. In our study, no significant difference in the risk for male-to-female HPV transmission as compared to female-to-male transmission was found and remarkable heterogeneity was found in subgroup analysis according to the carcinogenicity and species of the HPV in question. That is, the male to female transmission risk for oncogenic mucosal HPV was significantly higher than that from female to male, and for non-oncogenic HPV, the reverse of this result was observed. The studies by Nyitray *et al.*^6^ and Mbulawa *et al.*^7^ carried out analysis stratified by carcinogenicity in male-to-female transmission and female-to-male transmission separately, while the other three studies lacked the statistical power to do so. These two studies both concluded that the risk of female-to-male transmission was homogeneously higher in oncogenic and non-oncogenic HPV than that of male-to-female transmission. In western populations, the numbers of lifetime sexual partners in men and women are both relatively high, and little gender difference could be observed. That is, men and women should have equal chances to be the index side and transmit HPV to his/her partner through sexual contact. For example, in the study by Hernandez *et al.*, the mean number of lifetime sexual partners was 11.0 for men and 5.3 for women^10^. The study by Burchell *et al.* reported that the proportion of >5 lifetime sexual partners was 64.4% for men and 59.4% for women^8^. Widdice *et al.* also reported that the median number of lifetime sexual partners was 12.0 for men and 7.0 for women^9^. Nyitray *et al.* found the proportion of ≥3 lifetime sexual partners was 74.7% and 73.7% in men and women^6^. But in this rural China population, sexual behavior was much more conservative, and the difference in the levels of sexual conservativeness (multiple sexual partners) between men and women was much more pronounced (11.1% vs. 0.8%, p < 0.001).Thus, men are more likely to be the index partner in a given couple and transmit oncogenic HPV to their female partner. Women, the majority of whom remain monogamous, have served as “victims” of exogenous oncogenic HPVs in rural Chinese population. This is likely the major cause in the difference in oncogenic HPV transmission dynamics in these populations.

Another possible explanation was the similar prevalence but higher proportion of persistent infection in oncogenic HPVs in males as compared to females observed in our study (15.2% vs. 4.9%, *p* = 0.003). In the 5 published studies we evaluated for comparison, only Burchell *et al.* reported the proportion of “persistent” infection after 4 months, and estimates found of 59.5% for men and 60.6% for women were without significant gender difference^8^. Studies have shown that HPV persistence is correlated with high levels of viral load^29^,^30^,^31^. Thus, the longer exposure period and potentially higher exposure level to oncogenic HPV would theoretically lead to a higher risk for women acquiring oncogenic HPV from their husbands rather than vice versa in these couples. Our finding that more persistent infection existed in men is the reverse of that in Giuliano’s study in the US which reported men had more transient HPV infections than women^25^. However, it should be noted that the period sampling design in this type of study cannot efficiently distinguish persistent infection from repeated transient HPV infection due to the more active sexual behavior in men than in women. Further evidence from studies evaluating HPV variants and viral loads are required to validate this explanation.

Regarding cutaneous HPVs, a reverse trend was observed where the risk of male-to-female transmission was lower than that of female-to-male transmission. In this study, only cervical mucosa was sampled in women, while large regions of skin tissue were sampled in men. This may have led to underestimation of cutaneous HPV infection in women, which may explain why only 1/9 cutaneous HPV infections in women were detected, as compared to men. Thus, the likelihood of detecting a male-to-female cutaneous HPV transmission event would be much lower than female-to-male transmission.

For concordance estimates, a meta-analysis of 30 HPV type-specific concordance studies conducted in Europe or Asia^32^ reported an average estimate of HPV concordance of 25.5%, which is higher than our result (15.52%). Regarding transmission rate, studies by Mbulawa^7^ and Nyitray^6^, which were similar to our study in age range and study design also reported a higher HPV transmission rate (7.3-28.0 per 1000 PM) than we observed (5.56-7.11 per 1000 PM). Conservative sexual behavior in our population may be part of the reason for this discrepancy.

For the factor analysis, we found HPV concordance and transmission was more likely to occur in younger couples. Potential explanations for this trend include 1) younger couples are more sexually active, 2) older couples have longer periods of HPV exposure, and antibodies generated by previous infections may protect against reinfection by identical HPV types^28^. In the current study, we found that sex related variables, including number of lifetime sexual partners and sexual frequency, were positively associated with HPV concordance and transmission in couples, which is consistent with the study in the US by Hernandez^10^. Similar to Burchell’s study^8^, we also found persistent infection was the dominant indicator of likelihood of HPV transmission, and viral load may be the possible mechanism underlying this correlation of persistence and HPV concordance and transmission^19^,^29^,^30^,^31^.

Two limitations of this study should be noted. First, although we have investigated over 800 couples for as many as 7 visits, statistical power is still limited when analyzing specific types due to the low prevalence and transmission incidence of genital HPV infection in this population. Larger studies with greater numbers of cycles of evaluation are still needed to validate our conclusions. Secondly, as couples in the current study were not asked to avoid sexual intercourse for 48 hours before each visit, superficial virus deposited by sexual partners during recent intercourse may have confounded results. However this would likely not significantly impact our conclusions in view of the fact most couples reported having ≤1 episodes of sexual intercourse per week.

The results of our study indicate men in rural China play a much more important role than men in other populations as a source of genital oncogenic HPV infection. While anti-HPV intervention programs are already being planned for women to decrease the burden of HPV-related disease in this population, this further argues for the necessity of such programs in young and sexually active male adults. This is the first population-based prospective study employing a rigorous periodic sampling method to assess genital HPV transmission in a Chinese population. As no national HPV and cervical cancer screening program has been established in rural China, and in view of the fact prophylactic vaccines are still under clinical trial in China pending government approval, our findings may be of significance in targeting a high risk population for development of cost-effective vaccination-based cervical cancer and HPV screening programs.

## Additional Information

**How to cite this article**: Liu, M. *et al.* Transmission of genital human papillomavirus infection in couples: a population-based cohort study in rural China. *Sci. Rep.* **5**, 10986; doi: 10.1038/srep10986 (2015).

## Figures and Tables

**Table 1:** Selected demographic and behavior variables for genital HPV transmission in study couples from rural Anyang, China, 2009-2013.

		Male			Female	
Variable	Total N = 874	Included in concordance analysis N=406	Included in transmission incidence analysis N=296	Total N=874	Included in concordance analysis N=406	Included in transmission incidence analysis N=296
	n (%)	n (%)	n (%)	n (%)	n (%)	n (%)
Age at enrollment (years)						
Median (IQR)	44 (37-54)	44 (38-54)	44 (39-54)	44 (37-53)	44 (38-53)	44 (39-52.5)
≤45	505 (57.8)	239 (58.9)	167 (56.4)	507 (58.0)	237 (58.4)	165 (55.7)
>45	369 (42.2)	167 (41.1)	129 (43.6)	367 (42.0)	169 (41.6)	131 (44.3)
*P* value a	-	0.545^b^	0.560^c^	-	0.839^b^	0.331^c^
Education level						
Primary school or below	242 (27.7)	109 (26.8)	80 (27.0)	438 (50.1)	198 (48.8)	150 (50.7)
Junior middle. School	472 (54.0)	221 (54.4)	158 (53.4)	343 (39.2)	167 (41.1)	115 (38.9)
Above junior middle. school	110 (12.6)	53 (13.1)	43 (14.5)	53 (6.1)	30 (7.4)	26 (8.8)
Unknown	50 (5.7)	23 (5.7)	15 (5.1)	40 (4.6)	11 (2.7)	5 (1.7)
*P* value a	-	0.839^b^	0.492^c^	-	0.238^b^	0.080^c^
Job type						
Farmer	331 (37.9)	157 (38.7)	118 (39.9)	772 (88.3)	359 (88.4)	266 (89.9)
Worker in local area	261 (29.9)	121 (29.8)	90 (30.4)	49 (5.6)	24 (5.9)	18 (6.1)
Worker outside local area	264 (30.2)	124 (30.5)	84 (28.4)	24 (2.7)	13 (3.2)	8 (2.7)
Unknown	18 (2.1)	4 (1.0)	4 (1.4)	29 (3.3)	10 (2.5)	4 (1.4)
*P* value a	-	0.967^b^	0.612^c^	-	0.725^b^	0.941^c^
No. of lifetime sexual partners						
≤1	759 (86.8)	345 (85.0)	253 (85.5)	838 (95.9)	392 (96.5)	289 (97.6)
>1	97 (11.1)	57 (14.0)	39 (13.2)	7 (0.8)	4 (1.0)	3 (1.0)
Unknown	18 (2.1)	4 (1.0)	4 (1.4)	29 (3.3)	10 (2.5)	4 (1.4)
*P* value a	-	0.013^b^	0.179^c^	-	0.712^b^	0.643^c^
Persistent HPV infection						
No	-	342 (84.2)	243 (82.1)	-	387 (95.3)	283(95.6)
Yes	-	64 (15.8)	53 (17.9)	-	19 (4.7)	13(4.4)
*P* value a	-	-	-	-	-	-
Frequency of sexual intercourse (per week)						
≤1	441 (50.5)	211 (52.0)	151 (51.0)	527 (60.3)	251 (61.8)	183(61.8)
2	186 (21.3)	86 (21.1)	68 (23.0)	116 (13.3)	53 (13.1)	41(13.9)
>2	125 (14.3)	53 (13.1)	39 (13.2)	125 (14.3)	56 (13.8)	39(13.2)
Unknown	122 (14.0)	56 (13.8)	38 (12.8)	106 (12.1)	46 (11.3)	33(11.1)
*P* value a	-	0.557^b^	0.620^c^	-	0.818^b^	0.729^c^

^a^*P* values were derived from the Chi-square test.

^b^Comparison of couples included in concordance analysis (N = 406) and not included (N = 468).

^c^Comparison of couples included in transmission analysis (N = 296) and not included (N = 578).

**Table 2:** Concordance of HPV infection in 406 couples from rural China, 2009–2013*.

HPV type^a^	Infection of male	Infection of female	Total infection in couples	Observed concordant infection	Proportion of concordance & 95%CI (%)^b^	Expected concordant infection	*P* value^c^
**Any type**	327	192	406	63	15.52 (12.13-19.41)	9.32	<0.001
**Mucosal-oncogenic type**^d^	137	155	241	39	16.18 (11.77-21.45)	7.60	<0.001
HPV-16	64	65	115	14	12.17 (6.82-19.58)	5.36	<0.001
HPV-18	24	34	55	3	5.45 (1.14-15.12)	1.05	0.083
HPV-58	23	27	43	7	16.28 (6.81-30.70)	0.8	<0.001
HPV-45	13	6	15	4	26.67 (7.79-55.10)	0.1	<0.001
HPV-35	7	7	11	3	27.27 (6.02-60.97)	0.06	<0.001
HPV-33	6	16	20	2	10.00 (1.23-31.70)	0.12	0.006
HPV-52	4	11	13	2	15.38 (1.92-45.45)	0.06	0.001
HPV-68	6	5	9	2	22.22 (2.81-60.01)	0.04	<0.001
HPV-66	2	3	4	1	25.00 (0.63-80.59)	0.01	0.008
HPV-56	3	2	4	1	25.00 (0.63-80.59)	0.01	0.008
HPV-39	3	2	5	0	0	0.01	0.992
HPV-31	1	3	3	1	33.33 (0.84-90.57)	0	0.004
HPV-59	2	2	4	0	0	0.01	0.995
**Non-oncogenic type**^e^	252	55	269	28	10.41 (7.03-14.69)	1.75	<0.001
**Mucosal-non-oncogenic type**^f^	108	37	115	21	18.34 (11.69-26.52)	0.65	<0.001
HPV-54	24	6	26	4	15.38 (4.36-34.87)	0.19	0.007
HPV-90	17	2	17	2	11.76 (1.46-36.44)	0.04	<0.001
HPV-87	20	10	25	5	20.00 (6.83-40.70)	0.26	<0.001
HPV-67	13	2	13	2	15.38 (1.92-45.45)	0.03	<0.001
HPV-81	13	1	13	1	7.69 (0.19-36.03)	0.02	0.017
HPV-30	7	3	9	1	11.11 (0.28-48.25)	0.03	0.027
HPV-11	6	5	9	2	22.22 (2.81-60.01)	0.04	<0.001
HPV-6	6	4	7	3	42.86 (9.90-81.59)	0.03	<0.001
HPV-89	2	4	4	2	50.00 (6.76-93.24)	0.01	<0.001
HPV-74	2	2	4	0	0	0.01	0.995
HPV-42	2	1	3	0	0	0	0.997
**Cutaneous type**^g^	172	19	184	7	3.81 (1.52-7.70)	1.10	<0.001
HPV-3	92	4	93	3	3.23 (0.67-9.14)	0.47	0.006
HPV-57	50	9	57	2	3.51 (0.43-12.11)	0.58	0.110
HPV-43	7	2	8	1	12.50 (0.32-52.65)	0.02	0.018
HPV-91	7	1	7	1	14.29 (0.36-57.87)	0.01	0.009
HPV-10	8	1	9	0	0	0.01	0.990
HPV-75	4	2	6	0	0	0.01	0.990

^*^Couples were treated as the calculating unit.

^a^Only HPV types detected in both sexes were listed.

^b^Exact confidence intervals were used for specific types. 95% CIs for any HPV, oncogenic HPV and non-oncogenic HPV were calculated using binomial distribution.

^c^*P* values were calculated using the Chi-square test for specific types. For any HPV, oncogenic and non-oncogenic HPV, the Monte Carlo simulation method was used.

^d^Mucosal-oncogenic types included HPV-16,18,31,33,35,39,45,51,52,56,58,59,66 and 68.

^e^Non-oncogenic HPV included mucosal-non-oncogenic types and cutaneous types.

^f^Mucosal-non-oncogenic HPV types included HPV-2, 6, 11, 26, 30, 32, 42, 53, 54, 62, 67, 69, 70, 74, 81, 82, 83, 84, 87, 89 and 90.

^g^Cutaneous HPV included HPV-3,7,10,27,29,37,40,43,57,75,77,91 and 94.

**Table 3:** Transmission rate of HPV infection in 296 couples from rural China, 2009-2013.

	Male to Female		Female to Male			
HPV type^a^	Incident case	Incidence rate & 95%CI (/1000 PM)^b^	Incident case	Incidence rate & 95%CI (/1000 PM)^b^	Hazard ratio & 95%CI^c^	*P* value^c^
**Any type**	24	7.11 (4.81-10.93)	9	5.56 (2.98-11.56)	1.25 (0.56-2.78)	0.588
HPV-16/18^d^	5	7.71 (3.31-22.04)	1	1.46 (0.21-10.39)	4.91 (0.61-39.66)	0.135
HPV-6/11^d^	2	105.82 (47.59-292.78)	1	20.48 (2.88-145.37)	4.07 (0.42-39.53)	0.226
HPV-16/18/6/11^d^	7	10.48 (5.16-24.37)	2	2.73 (0.61-25.92)	3.69 (0.79-17.11)	0.096
**Mucosal-oncogenic type**^e^	13	12.13 (7.25-21.66)	3	2.37 (0.75-11.29)	4.89 (1.36-17.55)	0.015
HPV-16	5	10.91 (4.54-26.21)	1	2.52 (0.35-17.87)	3.95 (0.45-34.49)	0.215
HPV-58	2	11.84 (2.96-47.33)	1	4.88 (0.69-34.64)	3.20 (0.29-35.36)	0.342
HPV-45	0	0	1	37.55 (5.29-266.55)	-	-
HPV-35‘	2	38.41 (9.61-153.59)	0	0	-	-
HPV-33	1	40.21 (5.66-285.49)	0	0	-	-
HPV-52	1	154.64 (21.78-1097.80)	0	0	-	-
HPV-68	1	27.00 (3.80-191.69)	0	0	-	-
HPV-31	1	154.64 (21.78-1097.80)	0	0	-	-
**Non-oncogenic type**^f^	11	4.77 (2.72-9.13)	6	17.01 (7.72-42.60)	0.27 (0.10-0.76)	0.013
**Mucosal-non-oncogenic type**^g^	9	9.92 (5.41-20.06)	4	16.72 (6.31-54.47)	0.60 (0.19-1.90)	0.382
HPV-87	3	19.56 (6.31-60.65)	1	15.92 (2.24-113.04)	0.82 (0.07-9.42)	0.876
HPV-6	2	157.90 (39.49-631.33)	0	0	-	-
HPV-90	1	8.36 (1.18-59.37)	0	0	-	-
HPV-67	1	7.80 (1.10-55.35)	0	0	-	-
HPV-30	1	14.00 (1.97-99.38)	0	0	-	-
HPV-54	1	5.45 (0.77-38.70)	0	0	-	-
HPV-81	0	0	1	81.52 (11.48-578.73)	-	-
HPV-11	0	0	1	26.98 (3.80-191.52)	-	-
HPV-89	0	0	1	34.01 (4.79-241.47)	-	-
**Cutaneous type**^h^	2	1.43 (0.36-5.72)	2	17.62 (4.41-70.46)	0.05 (0.00-0.49)	0.010
HPV-3	0	0	1	41.78 (5.89-296.62)	-	-
HPV-57	1	2.34 (0.33-16.60)	1	15.48 (2.18-109.89)	NA	NA
HPV-91	1	22.42 (3.16-159.17)	0	0	-	-

^a^Only HPV types which showed transmission events in couples were listed.

^b^Confidence intervals were adjusted using the jackknife method for multiple infection in one subject in any HPV type, oncogenic HPV, non-oncogenic HPV, HPV-16/18, HPV-6/11 and HPV-16/18/6/11.

^c^Hazard ratios (M-F vs. F-M) and *P* values were derived from Cox models using the WLW method to account for within subject correlations in groups of HPVs.

^d^The slashes denote HPV transmission event of either one of these grouped types.

^e^Mucosal-oncogenic included HPV-16,18,31,33,35,39,45,51,52,56,58,59,66 and 68.

^f^Non-oncogenic HPV included mucosal-non-oncogenic types and cutaneous types.

^g^Mucosal-non-oncogenic HPV types included HPV-2, 6, 11, 26, 30, 32, 42, 53, 54, 62, 67, 69, 70, 74, 81, 82, 83, 84, 87, 89 and 90.

^h^Cutaneous HPV included HPV-3,7,10,27,29,37,40,43,57,75,77,91 and 94.

**Table 4:** Factors associated with concordance and transmission of genital HPV infection in couples from rural China, 2009-2013*.

	Concordance in couples^a^			Transmission in couples^b^		
Factor	Any HPV	Mucosal-oncogenic HPV^c^	Non-oncogenic HPV^d^	Any HPV	Mucosal-oncogenic HPV^c^	Non-oncogenic HPV^d^
	Adjusted OR (95%CI)	Adjusted OR (95%CI)	Adjusted OR (95%CI)	Adjusted HR (95%CI)	Adjusted HR (95%CI)	Adjusted HR (95%CI)
Age at enrollment (years)						
≤45	1.00	1.00	1.00	1.00	1.00	1.00
>45	0.58 (0.34-0.99)^e^	0.41 (0.20-0.84)^e^	0.84 (0.38-1.85)^e^	0.70 (0.35-1.41)^e^	0.81 (0.30-2.18)^e^	0.61 (0.21-1.76)^e^
Education level						
Primary school or below	1.00	1.00	1.00	1.00	1.00	1.00
Junior middle School	0.74 (0.35-1.57)	0.67 (0.27-1.71)	0.73 (0.26-2.10)	2.01 (0.62-6.52)	2.90 (0.59-14.24)	1.62 (0.32-8.25)
Above junior middle school	0.90 (0.37-2.19)	0.50 (0.15-1.74)	1.59 (0.45-5.66)	2.02 (0.57-7.21)	1.86 (0.28-12.58)	2.75 (0.50-15.18)
Job type						
Farmer	1.00	1.00	1.00	1.00	1.00	1.00
Worker in local area	1.51 (0.80-2.84)	0.96 (0.41-2.29)	3.01 (0.93-9.69)	1.09 (0.46-2.61)	0.89 (0.20-3.98)	1.63 (0.40-6.68)
Worker outside local area	1.80 (0.93-3.47)	1.32 (0.54-3.21)	3.02 (0.95-9.53)	1.11 (0.41-3.02)	0.46 (0.07-3.13)	2.39 (0.56-10.15)
No. of lifetime sexual partner						
≤1	1.00	1.00	1.00	1.00	1.00	1.00
>1	2.69 (1.60-4.53)	3.61 (1.68-7.76)	1.96 (0.87-4.41)	2.29 (1.07-4.90)	2.08 (0.63-6.87)	2.34 (0.92-5.95)
Persistent HPV infection						
No	1.00	1.00	1.00	1.00	1.00	1.00
Yes	6.34 (3.65-11.03)	11.16 (4.95-25.18)	4.02 (1.76-9.17)	2.25 (1.11-4.56)	6.95 (2.63-18.39)	0.66 (0.25-1.74)
Frequency of sexual intercourse (per week)						
≤1	1.00	1.00	1.00	1.00	1.00	1.00
2	2.84 (1.42-5.68)	1.99 (0.77-5.12)	5.92 (1.65-21.16)	2.39 (0.99-5.79)	1.38 (0.35-5.48)	4.78 (1.22-18.76)
>2	1.11 (0.36-3.42)	0.73 (0.19-2.78)	2.38 (0.50-11.45)	1.61 (0.57-4.57)	0.94 (0.19-4.66)	3.22 (0.65-15.90)

^*^Multivariate analysis of factors associated with HPV concordance and transmission. Each factor was adjusted for the average age of couples separately.

^a^GEE logistic regression models were used in concordance analysis.

^b^WLW Cox regression models were used in transmission analysis.

^c^Mucosal-oncogenic included HPV-16,18,31,33,35,39,45,51,52,56,58,59,66 and 68.

^d^Non-oncogenic HPV included mucosal-non-oncogenic types and cutaneous types

^e^Results of univariate regression models are presented for the variable “age”.
